# Effect of ASA on pituitary adenomas: is it a matter of survivin?

**DOI:** 10.18632/oncotarget.27054

**Published:** 2019-07-16

**Authors:** Fabio Rotondo, Kalman Kovacs

**Affiliations:** ^1^ Department of Laboratory Medicine, Division of Pathology and The Keenan Research Centre for Biomedical Science at the Li Ka Shing Knowledge Institute, St. Michael's Hospital, University of Toronto, Toronto, ON, Canada

**Keywords:** acetylsalicylic acid, cell cycle, pituitary adenoma, survivin

***News on:** Survivin as a potential therapeutic target of acetylsalicyclic acid in pituitary adenomas by Németh K et al. Oncotarget. 2018; 9:29180–29192. https://doi.org/10.18632/oncotarget.25650*

Since it was first synthesized by Felix Hoffmann in 1897, acetylsalicylic acid (ASA) has become the most widely used nonsteroidal anti-inflammatory drug (NSAID) in the world to treat a broad range of conditions including fever, pain and inflammatory disease. In the late 1980’s a case-control study was the first to demonstrate the negative correlation between ASA and colorectal cancer, suggesting that ASA may provide a protective effect against cancer [1]. Since then, various clinical and laboratory studies have revealed the anti-cancer effect of ASA in a variety of organs including colorectal, gastroesophageal, breast, lung, prostate, liver and skin cancers [2–4]. Although ASA seems to have both chemopreventive and anti-tumorigenic effects, the precise mechanism by which it exerts its effects has not yet been fully elucidated. It has been hypothesized that ASA may act on well- known cancer pathways to suppress progression and metastases. Various mechanisms for the induction of apoptosis by ASA have been proposed, including the activation of caspases or by inhibiting the enzyme cyclooxygenase (COX). In recent studies, ASA has been described to mediate the COX-independent apoptosis by down-regulating anti-apoptotic proteins [5].

Survivin, is a member of the inhibitor of apoptosis protein (IAP) family, which has been shown to play a role in regulating cell division and inhibiting apoptosis. Although, the molecular mechanisms of survivin regulation have not yet been fully elucidated, survivin is known to be overexpressed in various human cancers and fetal tissue, but is completely absent in normal adult tissue [6]. Since its expression is not detectable in normal tissue, it has emerged as a reliable target for not only tumor diagnosis and prognosis but also as a new target for anti-cancer therapy [6]. It was shown that its expression correlates with more aggressive tumors and poor clinical outcome [6]. Activation of apoptosis inhibiting signals has been shown to play a critical role in tumorigenesis, invasion and metastasis [7]. It has also been demonstrated that survivin can inhibit both caspase activity and apoptosis in tissue thereby working in contrast to the effects of ASA.

In the publication by Németh K et al., [8], they described the role of survivin in pituitary adenomas (PAs) as well as a new mechanism by which the apoptotic effect of ASA can be used to target PA cells. Representing approximately 20% of intracranial neoplasms, PAs are considered the third most frequent intracranial neoplastic tumor type, shadowing only meningiomas and glioblastomas [9]. They typically present with pituitary dysfunction and are usually discovered when they exert mass effects on surrounding tissues due to invasion into sphenoid sinuses and/or the sellar/parasellar regions leading to visual impairments, headache, and hypopituitarism. Morphologically, PAs represent a heterogeneous group of tumors and are classified as clinically functioning or silent (nonfunctioning) based on their clinical, radiological, and endocrinological findings, along with tumor size, and invasiveness [9]. Clinically nonfunctioning PAs (NFPAs) account for 33% of all PAs and are surpassed only by prolactin-producing PAs (47%). They pose a challenge to clinicians because they are mainly diagnosed incidentally as they are not typically associated with pituitary hormone hypersecretion/deficiencies and are usually asymptomatic until they present with clinical manifestations resulting from mass effect of the tumor. The oncogenic processes controlling the development of NFPAs are likely to involve multiple, simultaneously occurring irregularities in the control of the cell cycle. Previous studies have revealed that survivin expression in PAs was higher than in normal pituitary tissue thereby making survivin (over)expression a suitable marker for PAs [10].

The current study [8] is a useful and well-written addition to the literature involving antitumor effects of ASA via the survivin pathway. They demonstrated that from the various pituitary tissue types they analyzed, survivin was overexpressed only in the NFPAs. While ASA decreased cell proliferation, it did not induce apoptosis in pituitary cells and they noted that ASA could decrease cells in the S phase but increased cell number in the G2/M phase of a cell cycle. Its effect on cell cycle was the result of ASA’s ability to downregulate survivin as well as to inhibit cyclin A and CDK2. Authors also studied whether the effects of ASA-induced growth inhibition could be reversed by inhibiting survivin expression and found that growth inhibition could be only partially reversed if survivin was inhibited using either an inhibitor or SiRA-mediated silencing method. They concluded that because of the inhibition potential of ASA utilizing survivin inhibition as the key mechanism to deter PA tumor growth, it is a less expensive option compared to other chemotherapeutic treatment options to treat NFPAs. Obviously, more studies are needed to decide how effective the use of ASA and survivin inhibitors are as therapeutic options for treating not only NFPAs but all types of PAs but their novel study offers clinicians and surgeons new insights into PA aggressiveness as well as an early treatment regimen and a cheaper, effective and less invasive therapy for PAs.
